# A Robust Statistical Model to Predict the Future Value of the Milk Production of Dairy Cows Using Herd Recording Data

**DOI:** 10.3389/fvets.2017.00013

**Published:** 2017-02-17

**Authors:** Kaare Græsbøll, Carsten Kirkeby, Søren Saxmose Nielsen, Tariq Halasa, Nils Toft, Lasse Engbo Christiansen

**Affiliations:** ^1^Dynamical Systems, Department of Applied Mathematics and Computer Science, Technical University of Denmark, Lyngby, Denmark; ^2^Section for Epidemiology, National Veterinary Institute, Technical University of Denmark, Frederiksberg, Copenhagen, Denmark; ^3^Department of Large Animal Sciences, University of Copenhagen, Frederiksberg, Copenhagen, Denmark

**Keywords:** production parameters, modeling, simulation, prediction, lactation curve

## Abstract

The future value of an individual dairy cow depends greatly on its projected milk yield. In developed countries with developed dairy industry infrastructures, facilities exist to record individual cow production and reproduction outcomes consistently and accurately. Accurate prediction of the future value of a dairy cow requires further detailed knowledge of the costs associated with feed, management practices, production systems, and disease. Here, we present a method to predict the future value of the milk production of a dairy cow based on herd recording data only. The method consists of several steps to evaluate lifetime milk production and individual cow somatic cell counts and to finally predict the average production for each day that the cow is alive. Herd recording data from 610 Danish Holstein herds were used to train and test a model predicting milk production (including factors associated with milk yield, somatic cell count, and the survival of individual cows). All estimated parameters were either herd- or cow-specific. The model prediction deviated, on average, less than 0.5 kg from the future average milk production of dairy cows in multiple herds after adjusting for the effect of somatic cell count. We conclude that estimates of future average production can be used on a day-to-day basis to rank cows for culling, or can be implemented in simulation models of within-herd disease spread to make operational decisions, such as culling versus treatment. An advantage of the approach presented in this paper is that it requires no specific knowledge of disease status or any other information beyond herd recorded milk yields, somatic cell counts, and reproductive status.

## 1 Introduction

Herd managers cull cows to maintain optimal milk production and maintain herd profitability. For this reason, it is essential for herd managers to continually assess their cows to make timely and appropriate culling decisions.

Herd simulation models used to determine optimal disease-control strategies may benefit by including cow- and herd-specific information, as individual cows will differ in production performance and susceptibility to diseases, and hence their future milk production potential. To make appropriate disease control decisions, it is important to be able to accurately predict a cow’s future production. This can be achieved by including prediction algorithms within simulation models.

There exist several methods for estimating the future value and hence the optimal time to cull a dairy cow. Many are based on retention pay-off (RPO) values estimated using marginal net returns (MNR) or expanded to dynamic programming (1–4). RPO methods are appropriate for determining optimal culling times when complete information on incomes and expenses are included and the herd manager follows the recommendations of the model. This includes disease information and sundry costs. However, information about the health status of individual cows is often scarce, even in countries where individual cow-level health data are collected routinely, as in Denmark. Furthermore, herd managers are not solely motivated by economic incentives but also by habitual and social incentives (5), which may limit the enactment of optimal financial recommendations.

A disadvantage of dynamic programming is the dimensionality problem, in which the number of possible states of a cow grows exponentially with the number of state variables (i.e., production potential, parity, dry off week, and pregnancy) (4). Therefore, a concrete prediction of the future potential of a cow based on its characteristics (such as production and survivability) may provide a useful alternative for cow- and herd-specific decisions, and hence better meet the requirements and expectations of herd managers.

Here, we present a robust method for estimating the future average production (FAP) from any given point in the lifetime of an individual dairy cow. Using this approach, FAP is estimated every time new herd recording data becomes available allowing cows to be ranked according to their expected future profitability. In this way, individual cow culling decisions can be both timely and evidence based.

## 2 Materials and Methods

Data on milk production, somatic cell counts, and demographic data were obtained from the Danish Cattle database (hosted by SEGES P/S, Aarhus N, Denmark) from which 610 herds with Holstein dairy cows were randomly selected among approximately 3,000 Danish herds participating in milk recording. Cows in each of the selected herds were herd tested either 6 or 11 times per year. Herd testing involved the measurement of milk yield (in kilograms), milk fat (percent) and milk protein (percent), and somatic cell count (expressed as the number of somatic cells per milliliter of milk). Most (91%) of the selected herds had herd testing carried out on 11 occasions throughout the year.

Determining FAP consists of several steps to determine: the average milk production, somatic cell count, survival, and correlation structures between these measures at the herd level. Here, we present how the future values of milk yield and somatic cell count were predicted, and how these values were combined with the predicted lifetime, in order to give the expected future average production (FAP).

### 2.1 Milk Yield

The value of milk depends on the amount of milk produced per day as well as protein and fat content. Daily milk yield was quantified as the number of kilograms of energy-corrected milk [ECM (6)], defined as:

(1) ECM=milk(0.122fat+0.077protein+0.249)

where *milk* is milk in kilograms, *protein* is protein in percent, and *fat* is fat in percent, the unit of ECM is kilograms.

We required one parameter to describe the milk yield of every cow and preferably a parameter that was stable within and between lactations. To achieve this, individual cow lactation yields were fitted as value relative to the herd average 305-day milk yield lactation curve based on Ref. (7), but extended so that it accounted for day of conception. Curves were fitted for the first, second, and subsequent lactation periods per herd, as a function of days in milk (DIM):

(2) $$f_{\mathrm{ijk}}^{\text{ECM}}\left(\text{DIM}\right)=\alpha_{\mathrm{ij}}^{M}\;a_{\mathrm{jk}}^{M}\;{\text{DIM}}^{b_{\mathrm{jk}}^{M}}\;\text{exp}\left(-\left(c_{\mathrm{jk}}^{M}+I_{P}d_{\mathrm{jk}}^{M}\left(\text{DIM}-{\text{DIM}}_{P}\right)\right)\text{DIM}\right)$$

where each cow, *i*, is defined by a milk yield level $\alpha_{\mathrm{ij}}^{M}$ that is proportional to the average cow in lactation *j* in herd *k*. The parameters $a_{\mathrm{jk}}^{M}$, $b_{\mathrm{jk}}^{M}$, $c_{\mathrm{jk}}^{M}$, and $d_{\mathrm{jk}}^{M}$ describe the average cow lactation curve per lactation per herd. The superscript *M* denotes parameters describing milk production, and is included to differentiate with parameters later introduced with the superscript *C* which relate to individual cow somatic cell count. *I_P_* is the identity operator which is 1 if the cow is pregnant and 0 otherwise. DIM*_P_* is the day of pregnancy in units of DIM. This extension that includes the pregnancy date is important when evaluating lactations that are longer than 305 days. This was implemented by first fitting the herd parameters, that returns the average milk yield of a cow in the given herd, then fitting *α^M^* parameters for the individual cows.

Given the large variability in milk recordings for the individual cow, we decided that at least six data points per lactation per cow were required to fit individual levels of milk yield (*α*s). Of the first lactation cows, 90% had at least six samples taken per lactation. This parametrization reduced the description of the individual cow to a single intuitively understandable parameter (*α^M^*), which is the relative production level compared to the average cow in the same herd.

### 2.2 Somatic Cell Count

The somatic cell count (SCC) is an indicator of infection status in the dairy cattle, and Danish milk companies penalize the price paid for milk according to the level of SCC. We shall later include the level of SCC of the individual cow in the FAP. We use the total test day SCC (tSCC) given by *tSCC* = *SCC*⋅*milk* to model SCC. This method of modeling the somatic cell count as total somatic cells significantly reduced the variance of the residuals compared to modeling as somatic cells per milliliter (8). A one-parameter per cow model of the somatic cell count inspired by the Wilmink curve (9) was fitted as:

(3) $$f_{\mathrm{ijk}}^{\text{tSCC}}\left(\text{DIM}\right)=\text{exp}\;\left(\text{exp}\;\left(\alpha_{\mathrm{ij}}^{C}\;\left(a_{\mathrm{jk}}^{C}+b_{\mathrm{jk}}^{C}\;\text{DIM}+\text{exp}\;\left(-\text{exp}\;\left(c_{\mathrm{jk}}^{C}\right)\;\text{DIM}\right)\;d_{\mathrm{jk}}^{C}\right)-e\right)\right)$$

where $\alpha_{\mathrm{ij}}^{C}$ is the tSCC level of each cow *i*, relative to the average cow in lactation *j* in herd *k*, and similar to the milk yield the average cow is described by the herd-level parameters $a_{\mathrm{jk}}^{C}$, $b_{\mathrm{jk}}^{C}$, $c_{\mathrm{jk}}^{C}$, and $d_{\mathrm{jk}}^{C}$. The price of milk in Denmark is adjusted using the number of somatic cells per milliliter, therefore, we reverted back to SCC per milliliter whenever calculating the value of a cow.

### 2.3 Correlation Structures between Lactations

To be able to predict future production levels of individual cows, correlation structures of levels of milk yield and tSCC, *α^M^*, and *α^C^*, between lactations were determined by fitting a linear regression model. That is, the correlation, $\xi_{\mathrm{jlk}}^{M}$, for *α^M^* between lactations *j* and *l* in herd *k*, was determined as the regression coefficients as fitted by lm() using R (10). This means that if the correlation is 0.5, a cow, *i*, observed to produce 10% more than the average cow in lactation 1 will be predicted to produce 5% more than the average cow in lactation 2, $\left(\alpha_{\mathrm{ij}}^{M}-1\right)=\xi_{\mathrm{jlk}}^{M}\left(\alpha_{\mathrm{il}}^{M}-1\right)$.

### 2.4 Survival

To determine the likely future yield of a cow, we need to determine the probability of further lactation periods from any given lactation period in any given herd. The probability of further lactations is framed as survival to subsequent lactations which was determined by observing the fraction of cows producing milk in a subsequent lactation as a function of their milk level, *α^M^*, and pregnancy status. An individual cow’s survival probability was parameterized as a logistic function to make it more smooth and robust especially in the case of few data points where, e.g., spline fitting might give non-monotone results particularly in the situation where a farm have few cows with large DIM:

(4) $$S_{\mathrm{jky}}\left({\text{DIM}}_{!P}\right)=A-\frac{A}{{\left(1+\left(2^{\nu }-1\right)e^{-B\left({\text{DIM}}_{!P}-M\right)}\right)}^{1/\nu }}$$

where *S_jky_* takes a sigmoid form and the terms *A_jky_*, *B_jky_*, *ν_jky_*, and *M_jky_* are shape parameters (the subscripts *jky* are not included in equation (4) for ease of reading). The parameter DIM_!_*_P_* is the number of days in milk for non-pregnant cows. *S* represents the probability of a cow surviving a minimum of one further lactation, given that she is not pregnant at DIM_!_*_P_*. The subscripts indicate that a parameter is determined for all herds *k*, lactation periods, *j*, and is categorized by milk yield, *y*. The number of lactation periods fitted depends on the data available for each herd. The milk yield is divided into groups based on the relative yield *α^M^*.

The final estimate of survival *S_jk_* used in the model was based only on cows with a milk level, *α^M^*, above 1, meaning cows producing average or above average compared to the herd level. Cows with a milk level below 1 were observed to have a reduced probability of surviving to further lactation periods, but this may be due to selection bias by the herd manager or indication of other problems such as disease. By comparison cows with a milking level above 1 were observed to have a stable survival independent of further increase in milk level.

### 2.5 Future Yield

Appropriate predictions of the future milk yield of a dairy cow can be made by considering past and present production performance. Given that the milk yield and somatic cell count levels of a cow (*α^M^* and *α^C^*) change over time, we need to identify the optimal balance between past and present. So each recorded measurement of milk or SCC was evaluated against the average cow in that lactation in the same herd giving an *α^M^* or *α^C^* value, which is represented in the following by *x*. We, then, used exponential smoothing to continually update the prediction of $\tilde{x}$ over time:

(5) $${\tilde{x}}_{t}=\lambda x_{t}+\left(1-\lambda \right){\tilde{x}}_{t-1}$$

where *λ* is the smoothing factor, *x_t_* is our observed quantity at time *t*, and ${\tilde{x}}_{t}$ is the smoothed average at time *t*. The $\tilde{x}$ is both our best estimate of the present milk or SCC level of the cow and, therefore, also our best prediction of the future.

**Figure 1:**
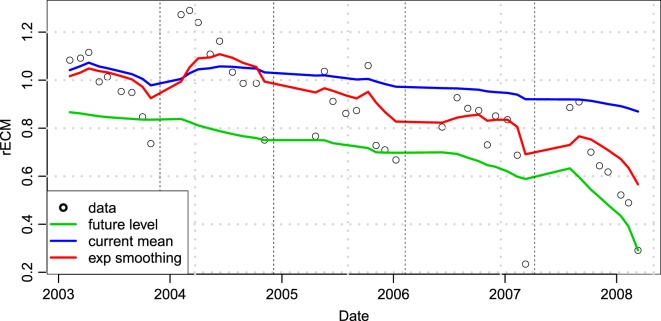
**An example of how smoothed average (red line) converges better to the future average (green line) compared to the mean of previous observations (blue line)**. Data points fitted are the relative milk yield (rECM) of an example cow compared to the herd average. Dashed lines indicate lactation periods. The example cow produces similar to the average cow in lactation one (rECM values around 1), above average in lactation 2, and then increasingly below average in subsequent lactations.

The level of smoothing as described by the parameter *λ* was estimated by taking the average over all future measurements ${\bar{x}}_{t>t_{n}}$ for all timepoints during the training period, *t_n_*, and finding the value of *λ* that minimized the squared difference between ${\tilde{x}}_{t_{n}}$ and ${\bar{x}}_{t>t_{n}}$ (see Figure 1).

As an initial value, ${\tilde{x}}_{0}$, we may use information about the yield of the dam. Where this information is not available, we use the value 1 to reflect that our best estimate is that the animal will be the same as the average animal in the herd.

### 2.6 Production Value of Milk

The previous sections describe the parameters required to predict the value of the future milk production of a dairy cow. Now we begin the process of combining this information into a single estimate of FAP.

First, we need to combine the ECM and the SCC to estimate the influence of the SCC on the profitability of the cow. There are several pricing corrections with regards to the SCC in Denmark. Here, we implement the version from the largest Danish milk processor, Arla Foods. Herd managers receive a corrected milk price based on the bulk milk somatic cell count. Prices for milk vary over time, and therefore, the correction schemes are determined in percentage. For this reason, we can consider a 10% reduction in the milk price as being equivalent to a 10% reduction in the milk yield.

The simplest implementation of price correction would be to take the SCC per measurement and directly apply the correction to the ECM produced by the individual cow. However, this method may overestimate the economic consequences, as an animal with a relatively high SCC would affect the price paid to the herd manager less in a large herd than in a small herd, because the SCC is diluted in the bulk tank.

Our proposed method emulates the removal of a single cow from the herd and determines the effect this will have on the bulk tank milk SCC. If removing a cow that produces more cells than are present in the bulk tank, this will change the bulk tank SCC by an amount proportional to the milk this cow produces. So the difference in SCC in the bulk, Δ*_c_*SCC*_B_* when removing cow *c* is given by:

(6) $$\Delta_{c}{\text{SCC}}_{B}=\frac{\sum_{i}^{i\neq c}\;{\mathrm{SCC}}_{i}\;{\mathrm{milk}}_{i}}{\sum_{i}^{i\neq c}\;{\mathrm{milk}}_{i}}-\frac{\sum_{i}\;{\mathrm{SCC}}_{i}\;{\mathrm{milk}}_{i}}{\sum_{i}\;{\mathrm{milk}}_{i}}$$

where we sum over all *i* cows milking on the same day in the same herd. This method provides an estimate of the contribution of cow *c* to the bulk tank somatic cell count.

**Figure 2:**
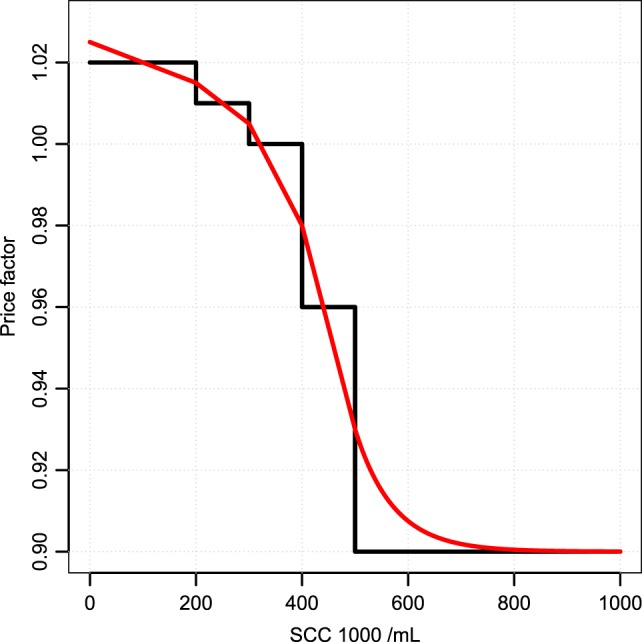
**Pricing interpolation**. How changes in the bulk milk SCC changes the value of milk. The black line is the pricing structure used by the largest Danish distributor. The red line is our interpolation.

Changes in the bulk tank milk from a specific cow are typically small and will not have economic consequences providing the typical structure, which is stepwise, changes in intervals of SCC (i.e., a bulk milk somatic cell count below 200,000 cells per milliliter gives a 2% increase in milk price, whereas a bulk milk somatic cell count between 400,000 and 500,000 cells per milliliter gives a 4% reduction in the milk price). Therefore, we interpolate and extrapolate the values in the pricing scheme so that all changes have an impact (see Figure 2).

The production value of milk (PVM*_c_*) from an individual cow, *c*, can now be calculated as:

(7) $${\text{PVM}}_{c}={\text{ECM}}_{c}+\left(f^{C}\left({\text{SCC}}_{B}\right)-f^{C}\left({\text{SCC}}_{B}+\Delta_{c}{\text{SCC}}_{B}\right)\;\right)\;\sum_{i}{\text{ECM}}_{i}$$

where ECM*_c_* is the energy-corrected milk production of the individual cow, *f^C^* is the interpolated price correction function in Figure 2, and $\sum_{i}\;{\text{ECM}}_{i}$ is the sum of lactating cows on a given day, equivalent to the total milk in the bulk tank. All further calculations will be measured in PVM, which has the unit of kilogram milk per day.

### 2.7 Future Average Production—FAP

The future average production is comprised of two parts: the value of milk from the reminder of the current lactation and the value of milk from future lactations.

#### 2.7.1 Value of Milk from the Remainder of the Current Lactation

The value of milk from the remainder of the current lactation can be predicted for two categories of cows: those that are pregnant and those that are non-pregnant. The methods for calculating their values are similar, yet the end points of the lactation are determined in distinct ways. If a positive pregnancy test has been recorded, then the previous insemination attempt is regarded as the day of pregnancy, DIM*_P_*. For these cows, the last day of the lactation, DIM*_L_*, is set to:

(8) $${\text{DIM}}_{L}={\text{DIM}}_{P}+282-56$$

If the cow has not yet tested pregnant, the length of the lactation period will be set to:

(9) $${\text{DIM}}_{L}=\text{max}\left(\text{DIM}+282-56;61+282-56\right)$$

In equations (8) and (9), 282 days equals the average gestation period for Holstein cows, the dry period is 56 days, and the average time before achieving pregnancy is 61 days.

After calculating DIM*_L_* for each cow, the estimate for the milk and SCC levels (the $\tilde{\alpha }$s) are calculated using equation (5). For a first lactation dairy cow with no prior test records, the $\tilde{\alpha }$s are given by the correlation with the $\tilde{\alpha }$s of the dam. Using these values and equations (2) and (3), the milk and SCC production of the remainder of the current lactation is calculated. These values are used to calculate PVM values using an equation similar to equation (7), but using the fitted values from the training data.

(10) $$f_{\mathrm{ijk}}^{\text{PVM}}\left(t,{\tilde{\alpha }}^{M},{\tilde{\alpha }}^{C}\right)=f_{\mathrm{ijk}}^{\text{ECM}}\left(t,{\tilde{\alpha }}^{M}\right)+\left(f^{C}\left({\text{SCC}}_{B}\right)-f^{C}\left({\text{SCC}}_{B}+\Delta_{c}{\text{SCC}}_{B}\left(f_{\mathrm{ijk}}^{\text{tSCC}}\left(t,{\tilde{\alpha }}^{C}\right)\right)\right)\right)\sum_{i}{\mathrm{ECM}}_{i}$$

We note that while we use the predicted values of the individual cow for milk and SCC, we assume that the total amount of milk and the bulk SCC from the herd remains constant, as it would otherwise be necessary to also predict these future values, which would be complicated. The predicted future production of the current lactation, FP*_C_*, of a cow is therefore:

(11) $${\text{FP}}_{C}=\sum_{t=\text{DIM}}^{{\text{DIM}}_{L}}f_{\mathrm{ijk}}^{\text{PVM}}\left(t,{\tilde{\alpha }}^{M},{\tilde{\alpha }}^{C}\right)$$

For simplicity, we have removed the subscripts *ijk*, but FP*_C_* is dependent on the cow, the lactation, and the herd. The predicted future average production for the remaining current lactation is then:

(12) $${\text{FAP}}_{C}=\frac{{\text{FP}}_{C}}{{\text{DIM}}_{L}-\text{DIM}+56}=\frac{{\text{FP}}_{C}}{T_{\mathrm{RC}}}$$

where *T_RC_* equals the total time remaining in the current parity. We add the 56 days because the average production over time should also include the dry period.

#### 2.7.2 Value of Milk from Future Lactations

The value of future lactations is calculated in a similar way to that of the current lactation. However, here we use a standardized lactation length and the predicted value of the milk and SCC levels from equation (5) that were pre-calculated for the FAP*_C_*. We then use the fitted correlations of the milk and SCC levels between lactations, ξ. We can predict the PVM given the predicted ECM and SCC in a similar way to equation (10).

(13) $${\text{FP}}_{\mathrm{jlF}}=\sum_{t=3}^{{\text{DIM}}_{L}}f_{\mathrm{ijk}}^{\text{PVM}}\left(t,\xi_{\mathrm{jl}}^{M}{\tilde{\alpha }}^{M},\xi_{\mathrm{jl}}^{C}{\tilde{\alpha }}^{C}\right)$$

In equation (13) DIM*_L_* = 61 + 282 − 56 = 287, which is a simplification of equation (9), but this average lactating period could also be inferred from data for the individual herd. We keep only the subscripts *jl* as we must specify the lactation period being considered, from lactation *j*, to future lactation period, *l*.

(14) $${\text{FAP}}_{\mathrm{jlF}}=\frac{{\text{FP}}_{\mathrm{jlF}}}{282+61}=\frac{{\text{FP}}_{\mathrm{jlF}}}{T_{S}}$$

where *T_S_* is the standard time in a parity.

#### 2.7.3 FAP

Individual cow FAP estimates used for decision-making are calculated by weighing the future production with the different probabilities of surviving from the present lactation [equation (4)] to possible future lactations:

(15) $$\text{FAP}=\left(1-S_{\mathrm{jk}}\right)\;{\text{FAP}}_{C}+S_{\mathrm{jk}}\sum_{l=j+1}^{n}\gamma_{\mathrm{jlk}}\;\frac{{\text{FP}}_{C}+\sum_{m=j+1}^{l}\;{\text{FP}}_{\mathrm{jmF}}}{T_{\mathrm{RC}}+\left(l-j\right)\;T_{S}}$$

where *γ_jlk_* is the fraction of cows surviving from lactation period *j* to lactation period *l* in herd *k*.

Explanations of each of the acronyms used in these calculations and typical values of parameters are provided in Table 1. In Table 2 we present a step-by-step description of the process of calculating FAP.

**Table 1:** **Symbols and typical values**.

Index *i*	Individual cow	NA
Index *j*	Lactation	NA
Index *l*	Future lactation	NA
Index *k*	Herd	NA
$\alpha_{\mathrm{ij}}^{M}$	ECM level relative to average cow	N(1, 0.4)
$\alpha_{\mathrm{ij}}^{C}$	tSCC relative to average cow	N(1, 0.4)
*a*, *b*, *c*, *d*	Parameters describing fitted curves	–
*S_jky_*	Survival probability	0–1
*A*, *ν*, *M*	Parameters describing fitted curves	–
*λ*	Smoothing parameter	0.2
SCC*_B_*	Somatic cell count in the bulk tank (×1,000/mL)	100–500
Δ*_c_*SCC*_B_*	Change in SCC*_B_* when removing one cow	Equation (6)
PVM	Production value milk	Equation (7)
FP*_C_*	Future production of current lac.	Equation (11)
T*_RC_*	Time remaining of current lac.	Equation (11)
FAP*_C_*	Future average production of current lac.	Equation (12)
FP*_jlF_*	Future production of lac. *l*	Equation (13)
FAP*_jlF_*	Future average production of lac. *l*	Equation (14)
T*_S_*	Time of standard length lactation	321
ξ*_jl_*	Correlation between lactations	0.3
*γ_ijk_*	Fraction of survival	0–1

*Description of some of the symbols used and their typical values*.

**Table 2:** **Steps in FAP**.

Step	Description	Equations	Expressions
1	For each herd and lactation, fit parametric functions of ECM and SCC	(1)–(4)	ECM, tSCC
2	Express ECM and tSCC levels relative to average cow on the herd	(3) and (4)	*α^M^*, *α^C^*
3	For each herd and lactation, fit survival and correlations	(5) and (6)	*S*, *γ*, *ξ*, *λ*
4	Determine most likely current level of ECM and tSCC by exponential smoothing	(6)	*λ*
5	Predict future values of ECM and tSCC using the current level + correlation	(3)–(6)	*λ*, *α^M^*, *α^C^*
6	Assign probabilities of surviving to different lactations given repro status	(5)	*S*
7	Combine ECM and SCC to PVM for the different expected life courses	(7) and (8)	PVM
8	Calculate expected FP of PVM for each life course	(9)–(12) and (14)	FP
9	Average FP with expected lifetimes, and weight with survival probabilities to get FAP	(13), (15) and (16)	FAP

*Description of the steps needed to calculate the future average production value for every time data are updated*.

### 2.8 Validation of the Methodology

A subset of the herd recording data was taken to include only milk records for cows born after January 1, 1990. The model was trained on data before January 1, 2010, and FAP estimates were produced for the test period January 1, 2010 to January 1, 2013. Herds were excluded if they did not have an average of at least 50 lactating cows per milk recording date during the training period. This reduced the number of test herds to 388 herds.

Summary statistics of the included herds are provided in Table 3. We compared the FAP estimates calculated using the method described in this paper with a non-parametric approach which involved taking the mean of all future PVM for each cow. The future PVM was weighted with (281 + 61 − 56)/(281 + 61) to account for cows being dried off. The mean of future PVM measurements was compared to FAP, the last measured PVM, the mean of the last three PVM, and the lifetime mean of PVM for each cow.

**Table 3:** **Summary statistics of herds used to inform the FAP model**.

	Median	Range (2.5–97.5%)	Unit
Lactating cows per recording date	93	58–182	no.
Lactating cows total	854	470–2,081	no.
Prop. cows in lac. period 1	0.40	0.32–0.48	–
Prop. cows in lac. period 2	0.28	0.25–0.31	–
Prop. cows in lac. period 3	0.17	0.14–0.18	–
Prop. cows in lac. period ≥ 4	0.15	0.09–0.25	–
Avr. ECM lac. period 1	25.7	21.6–29.9	kg
Avr. ECM lac. period 2	29.1	24.0–34.7	kg
Avr. ECM lac. period 3	30.3	24.9–36.0	kg
Avr. ECM lac. period ≥ 4	30.0	24.7–36.2	kg

*All measures are calculated per herd and then summarized in this table*.

*Prop., proportion; lac., lactation; Avr., average; ECM, energy-corrected milk*.

Statistical comparisons were performed, unless otherwise stated, using the Wilcoxon rank sum test with continuity correction. All statistical analyses were carried out using R version 3.1.1 (10).

## 3 Results

**Figure 3:**
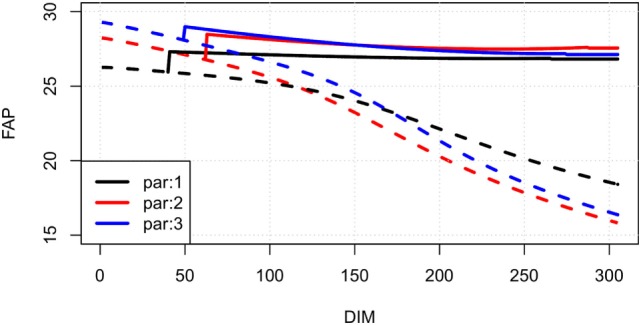
**Examples of FAP values of an average dairy cow in different parities on a randomly selected herd**. Solid lines are pregnant cows, dotted lines are non-pregnant. Colors describe FAP in different parities of cows (black: first parity, red: second parity, blue: third parity).

**Figure 4:**
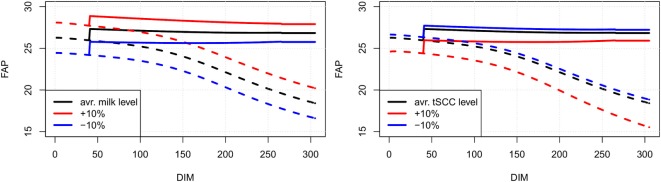
**Examples of FAP values for a first parity cow with varying milk (left) and SCC (right) levels (${\tilde{\alpha }}^{\text{M}}$ and ${\tilde{\alpha }}^{\text{C}}$)**. Solid lines are pregnant cows, dotted lines are non-pregnant. An increased milk production leads to a higher value of FAP (left), while increase in SCC leads to a decrease in FAP value (right). Colors represent change in $\tilde{\alpha }$^*M*^ and $\tilde{\alpha }$^*C*^ compared to the average cow (black: no change, red: increased by 10%, blue: decreased by 10%).

Line plots showing our estimates of FAP as a function of days in milk for an average cow of parities 1, 2, and 3 in a randomly selected herd are shown in Figure 3. In Figure 4 (left), FAP as a function of days in milk is shown for a parity 1 cow with herd average yield, ${\tilde{\alpha }}^{M}$, herd average yield less 10%, and herd average yield plus 10%. Similarly, in Figure 4 (right) FAP as a function of days in milk is shown for a parity 1 cow with herd average total somatic cell count level, ${\tilde{\alpha }}^{C}$, herd average total somatic cell count level less 10%, and herd total somatic cell count level plus 10%. First parity cows typically have a lower yield than higher parities; therefore, the FAP is lower in the beginning of the lactation. Later in each lactation, non-pregnant first parity cows have a higher survival probability, and therefore a higher FAP.

**Figure 5:**
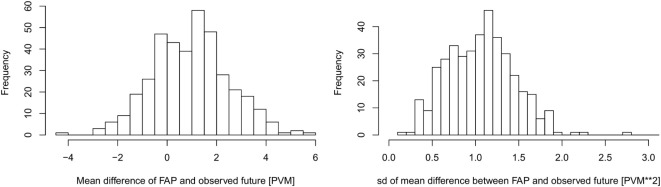
**The average difference (left) and SD (right) between FAP and the observed future (weighted) values of production value milk (PVM) at herd level**. The PVM is in units of kilogram milk with a percentage correction given by the level of somatic cells in the bulk milk (see Figure 2). Number of farms in the comparison *n* = 388.

The overall predictive value of FAP was estimated by comparing the predicted FAP of each dairy cow for each test day in the test dataset, compared to the known future value as described in the section 2.8. The results were averaged within herds, as shown in Figure 5.

In the majority of herds, FAP predicted future PVM within ±2 kg milk per day of the observed values. The SDs of the predictions were also estimated for each herd (Figure 5), which shows that the variance of FAP is small.

When comparing the prediction of FAP with the latest value of PVM, the FAP predictions were significantly better than future PVMs compared to the latest PVM (*P* = 0.013). The FAP was on average 50% closer to the future values compared to the latest PVM and more than 70% closer to the future values compared to the average of the latest three and lifetime mean (significantly different with *P* < 10^−4^). The SDs of the difference between FAP and mean of future PVM were also significantly smaller than that for the difference between the latest PVM and the mean of latest three PVM (*P* < 10^−8^). The difference between FAP and the lifetime mean showed similar SD.

## 4 Discussion

Even though replacement models have been available since the 1960s (11) and the theory behind optimal replacement using RPOs was fully formed in the 1980s (1, 2), few herd managers use these models. One reason for this lack of uptake may be that optimized culling using RPO models require herd managers to exercise rational behavior. However, the behavior of individual herd managers is known not to be always rational, but is instead frequently governed by a range of other factors including prior beliefs, values, attitudes, knowledge, and social norms (12). The FAP model is data driven and adaptive so that a change in herd manager behavior is reflected in the FAP values. Changing culling strategy will change the survival curve, changing feed will likely change lactation curves, both of these changes would affect the FAP value. Furthermore, we propose that the FAP is a more readily understood metric for herd managers (since it is expressed in monetary units) compared with RPOs, which are presented to herd managers using metrics that are conceptually more difficult to understand.

The FAP is designed as a tool to support herd managers in making culling decisions on the basis of production data only. We have shown that FAP can accurately predict the future production of milk and SCC combined into a single value, based on herd test information only.

Most herd managers will attempt to inseminate first parity cows for a longer time than cows in later lactations. This behavior leads to first parity cows having a higher survival in the high DIM when open compared with later parity cows. This results in a higher FAP value for first parity cows, as shown in Figure 3. The number of additional times a herd manager will inseminate first parity cows compared with cows in later parities can be herd-specific, and therefore, the survival curves vary among herds.

The presented FAP calculation methodology can be improved and extended. The bias shown in Figure 5 is primarily driven by not knowing the exact values of factors that may vary between herds, such as the length of the dry period, as well as the average number of days from calving to conception. We expect that if such information were available or inferred from data, our FAP estimates may be improved. Thus, further information can be added where it is specifically known. Other factors that may be included in order to improve the model could be seasonal variation both on the day of milking and the day of calving. Factors related to diseases that influence the milk yield may also be included. However, it would then be necessary for herd managers to consistently check their stock for disease and to consistently record the presence of disease.

The genetic value of a cow and its potential future calves could also possibly be included in the FAP. There are several genetic indexes in Scandinavia, an example of which is the Nordic Total Merit index (13). Furthermore, there are many different genetic indexes worldwide [e.g., Ref. (14)], and such information will often enter into the decision process to cull an animal by the herd manager. It is possible that genetic indexes with predictions based on observed values (such as the FAP) are most valuable to a herd manager, but this is dependent on individual management practices.

If large amounts of data are available (e.g., daily measurements of milk production), it may be desirable to track the production using more sophisticated methods such as a Kalman filter, that predicts optimally based on the resent measurement and the observed noise in previous measurements (15). However, the herd recording data used in this study (including milk production and SCC) is collected for individual cows on an almost monthly basis and is, therefore, relatively sparse. For this reason, we opted for the method of exponential smoothing. This method is more robust, less computationally demanding, does not require initialization, and is also less sensitive to non-uniform variation of data over the lactation period.

The FAP is a framework to estimate future production and is as such not dependent on the specific parametrization of the lactation or SCC curves. Therefore, it is possible to use this framework with any lactation or SCC curve desired, though the number of parameters per herd and per cow should be considered when choosing formulations of these curves.

Surprisingly, the latest PVM (which is the most recent observation of the cow) was the second best predictor of the future value after FAP. The latest PVM was better at predicting the future than the average of the last three measurements or the lifetime mean. However, given that milk yield typically decreases toward the end of a lactation, we suspect that the precision of the latest PVM may be most precise in the beginning and more uncertain toward the end of a lactation.

Theoretically, the optimal model for culling is the RPO, which requires full economic parameters (1, 4). However, there are large differences between herds for both income given by production values and expenses dependent on many parameters, including herd type, feeding strategies, and milking systems. We also note that many RPO methods include single diseases or production only, most likely due to the dimensionality problem and hence the high computational power demanded by these methods. However, not including all diseases such as mastitis, lameness, and udder malfunction may overestimate the maximum average value of a cow in the herd, leading to non-optimal culling decisions. Diseases are implicitly implemented in FAP, given that the milk production is continually updated *via* the exponential smoothing algorithm. However, we would expect correlation structures and survival to be different for diseased animals, and this information could be explicitly implemented if such information were available. Therefore, further improvements may be made by investigating how correlation structures for cows with diseases such as mastitis or paratuberculosis differ from healthy cows. Once such information is available, FAP can, in principle, include several diseases, with only little additional computational power required. This would make it possible to implement FAP within-herd-specific models in order to predict the future potential of individual cows for operational decisions, such as treatment and culling, while also taking into account the general strategic management within the herd. This will allow cow- and herd-specific decisions to be made, and is expected to simultaneously improve animal health and herd economy following implementation.

Another way of utilizing FAP predictions would be to compare marginal net production with the average healthy cow, not the optimal cow. This would allow a herd manager to compare the expected lifetime milk yield of given cow with that of a replacement.

## 5 Conclusion

We have shown how to predict the average future value corrected production FAP, which may be a useful option for making informed culling decisions for dairy cows, given sparse production and disease data and incomplete economic information. The FAP can be used on a day-to-day basis to directly rank cows for culling, or can be implemented in simulation models of disease spread to make inferences on operational decisions, such as culling and treatment, while examining the impact of management strategies of the herd. This is expected to improve animal health and dairy herd profitability.

## Author Contributions

Developed models, performed data analysis, wrote manuscript: KG. Assisted in developing of models, analysis of data, and drafting of manuscript: LC. Assisted in analysis of data and drafting of manuscript: CK, SN, TH, and NT.

## Conflict of Interest Statement

The authors declare that the research was conducted in the absence of any commercial or financial relationships that could be construed as a potential conflict of interest.
